# Behaviour change strategies to influence antibiotic prescribing in acute care: a systematic review

**DOI:** 10.1186/1753-6561-5-S6-O43

**Published:** 2011-06-29

**Authors:** E Charani, R Edwards, N Sevdalis, B Alexandrou, E Sibley, D Mullet, BD Franklin, A Holmes

**Affiliations:** 1 Centre for Infection Prevention and Management, Imperial College London, London, UK; 2 Deaprtment of Surgery and Cancer and Imperial Centre for Patient Safety and Service Quality, Imperial College London, London, UK; 3 Dr Foster Intelligence, London, UK; 4 Centre for Medication Safety and Service Quality, Imperial College Healthcare NHS Trust, London, UK

## Introduction / objectives

Antibiotic usage in acute care is widely reported to be suboptimal. Inappropriate use of antibiotics is a major contributing factor to emergence of multi-drug resistance and healthcare associated infection. Addressing antibiotic prescribing behaviour (APB) is a key component of antibiotic stewardship.

## Methods

We carried out a novel systematic review of both qualitative and quantitative literature on APB in acute care. We assessed the extent to which behavioural sciences and social marketing were applied and whether this could be related to the effectiveness of reported outcomes. MEDLINE, EMBASE, ASSIA, Business Source Complete, The Cochrane Library, PsycINFO, Dare and HMIC were searched for studies undertaken in 1999-2009 and published in English.

## Results

5 qualitative and 5 quantitative studies out of a total of 180 met the quality criteria. Qualitative studies highlight the predominant influence of social norms, attitudes, and beliefs on APB. Quantitative studies reporting interventions to optimise antibiotic prescribing do not use theoretical science or primary research to inform the design and choice of the interventions deployed.

## Conclusion

Despite qualitative evidence demonstrating the impact of behavioural determinants and social norms on prescribing, these influences are not given due consideration in the design and evaluation of interventions. To ensure a better understanding of APBs and to improve the quality of interventions and research in this area, the application of behavioural sciences supported by appropriate multidisciplinary collaboration is recommended.

## Disclosure of interest

None declared.

